# Somatosensory Mismatch Negativity in Children: A Narrative Review of Current Evidence and Methodological Considerations

**DOI:** 10.3390/diagnostics16101471

**Published:** 2026-05-12

**Authors:** Adelina Amalia Ardelean, Laura Alexandra Nussbaum, Andrei Brînzeu

**Affiliations:** 1 Department of Neurosciences, Neuroscience Research Center, Victor Babes University of Medicine and Pharmacy, 300041 Timisoara, Romania; 2 NeuroPain Lab, INSERM U1028, UMR5292, Lyon Neuroscience Research Center (CRNL), Claude Bernard University Lyon 1, 69500 Lyon, France; 3 Center for the Evaluation and Treatment of Pain (CETD), Neurological Hospital Pierre Wertheimer, Hospices Civils de Lyon, 69500 Lyon, France

**Keywords:** somatosensory mismatch negativity (sMMN), event-related potentials (ERP), pediatric neurodevelopment, sensory processing

## Abstract

Somatosensory mismatch negativity (sMMN) constitutes an electrophysiological marker that initially reflects preattentional processing and subsequently indexes automatic somatosensory deviance detection. While its application in adult populations is gradually expanding, the establishment of a standardized and reproducible methodology for eliciting and analyzing sMMN in pediatric populations remains uncertain. To determine whether the published literature provides a clear, consistent, and standardized methodology for sMMN assessment in individuals under 18 years. A search was conducted in PubMed (11), Scopus (6), Web of Science (6), DOAJ (1), Europe PMC (6), Embase (0), ClinicalKey (0), Cochrane Library (0) and ClinicalTrials.gov (0) database from inception to 18 August 2025. Eligible studies included research assessing sMMN using somatosensory oddball paradigms in participants <18 years. Inclusion criteria (participants younger than 18 years, the use of the oddball paradigm to evaluate somatosensory mismatch negativity, and clinical reports, single-case reports or experimental studies including both typically developing children and children with neurological conditions, published in English), and exclusion criteria (exclusivity for adult participants, and narrative reviews or editorials) were defined a priori, as well as the screening procedures and quality assessment methods. Two reviewers independently performed study selection and data extraction, with a third reviewer resolving disagreements. Risk of bias was assessed using the MMAT (mixed methods appraisal tool). Due to substantial heterogeneity in paradigms and outcome reporting, results were synthesized narratively. Four studies met inclusion criteria. Methodological diversity was pronounced across everything except task type that was passive. There is not a consensus regarding stimulation parameters. Risk of bias assessment revealed frequent concerns related to incomplete reporting and variability in analytic choices. The small number of studies, inconsistent methodological reporting, and absence of harmonized protocols limited comparability. Current evidence does not support the existence of a standardized methodology for assessing sMMN in children. Future studies should adopt harmonized stimulation paradigms and transparent, reproducible reporting standards to enable cross-study comparability and clinical translation.

## 1. Introduction

**Figure 1:**
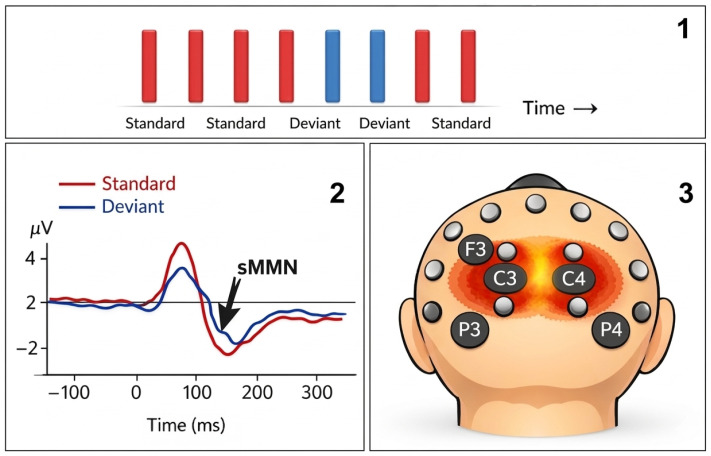
**1. Schematic illustration of the oddball paradigm (up)** used to study Mismatch Negativity (MMN). A sequence of repetitive stimuli (“Standards”, shown in blue) is occasionally interrupted by different stimulus (“Deviant”, shown in red). The brain’s automatic response to these deviant stimuli, compared to the standard ones, reflects the neural detection of change in the environment. **2. Waveform Graph (Down-Left Side)** Red Line: represents the cortical response to standard stimuli, serving as a baseline for brain activity when predictable tactile inputs are presented. Blue Line: represents the cortical response to deviant stimuli, i.e., unexpected or different inputs that trigger a surprise response. sMMN Peak (black arrow): indicates the point where somatosensory mismatch negativity occurs. This negative ERP component reflects the brain detecting a mismatch between the expected and actual stimulus. Axes: X-axis (“Time (ms)”): time from stimulus onset in milliseconds. Y-axis (“Amplitude (µV)”): cortical response amplitude in microvolts. The difference between the red and blue lines at the sMMN peak clearly shows where the brain detects the tactile deviance. **3. Scalp Map (Down-Right Side)**—top-down view of the head showing electrode positions. Highlighted areas: C3 and C4 (central left and right)—the primary sites where sMMN amplitude is most pronounced. Adjacent parietal electrodes (P3, P4) and frontal electrodes (F3) also show activation, but less strongly. Red–orange coloring: indicates scalp regions with the largest amplitude changes associated with sMMN.

The nervous system continuously monitors sensory input for events that deviate from ongoing regularities. Such unexpected stimuli tend to attract attention because they may signal biologically relevant change in the external environment or within the body itself. At the neurophysiological level, this capacity for automatic change detection is indexed by mismatch negativity (MMN), an event-related potential (ERP) typically elicited in oddball paradigms, in which infrequent deviant stimuli are embedded within a sequence of repetitive standard stimuli [1,2]. MMN is usually derived by subtracting the ERP response to standard stimuli from that elicited by deviant stimuli, yielding a negative deflection that generally occurs approximately 100 to 250 ms after stimulus onset [3]. This MMN response is most commonly recorded using scalp electroencephalography (EEG), through the placement of electrodes on the scalp. Depending on the specific type of MMN being investigated, the distribution of EEG activity allows researchers to infer the cortical regions involved in processing the deviant stimuli [4]. The topographical patterns of the recorded signals provide indirect evidence of the underlying brain areas activated during automatic change detection as seen in Figure 1. Importantly, MMN can be recorded even when attention is directed elsewhere, and is therefore considered a marker of automatic, pre-attentive sensory discrimination [5,6].

MMN has been demonstrated across several sensory modalities. Auditory MMN is the most extensively investigated [3,7,8], while visual MMN is also well established [9,10]. By contrast, somatosensory mismatch negativity (sMMN) has received comparatively limited attention and remains methodologically more difficult to study. The ability to register and prioritize such events is fundamental for adaptive behavior, as it contributes not only to basic sensory processing, but also to sensorimotor integration, body representation, and higher-order cognitive functioning [11,12,13]. In this regard, the study of sMMN offers a unique window into how the brain automatically processes bodily signals before conscious attention is engaged [14].

A developmental perspective is especially important in this context [15]. The neural mechanisms supporting sensory discrimination, attentional orienting, and body-related processing undergo substantial maturation from infancy through adolescence. Accordingly, the characteristics and functional significance of sMMN in children cannot be assumed to mirror those observed in adults, nor to remain stable across pediatric age groups. Existing studies suggest that automatic detection of somatosensory novelty is present from early stages of development [11]. Work in infants further indicates that sMMN may reflect not only deviance detection per se, but also more complex processes related to the emergence and delineation of body representations [12,13]. In parallel, intracranial and electrophysiological investigations in pediatric neurological populations have contributed to the characterization of the neural substrates underlying somatosensory discrimination [16].

A better understanding of sMMN in childhood may also have important clinical implications. Altered pre-attentive sensory processing has been implicated in neurodevelopmental conditions such as autism spectrum disorder [17,18] and attention-deficit/hyperactivity disorder, while somatosensory paradigms may also prove informative in neurological conditions including epilepsy, severe brain injury [19,20,21] and disorders of consciousness [22]. Despite this potential, the pediatric sMMN literature remains sparse and methodologically heterogeneous. Studies differ with respect to participants’ ages, stimulation parameters, deviant characteristics, recording procedures, and analytic approaches, making cross-study comparison difficult and limiting firm conclusions regarding developmental trajectories.

The present work was undertaken to clarify the current state of knowledge regarding sMMN in children across ages, with particular attention to evidence for its existence and its evolution during development. Although the literature search and study selection were conducted according to PRISMA principles, the limited number and heterogeneity of eligible studies did not permit a fully systematic synthesis. We therefore conducted a narrative review with systematic intent. By critically examining the available evidence, we sought to identify convergent findings, evaluate the robustness of current claims, and outline priorities for future research, including the need for clearer, more standardized, and reproducible methods for measuring sMMN in pediatric populations.

## 2. Objectives

**Primary objective:** To summarize and describe the electrophysiological characteristics of sMMN in children.

**Secondary objectives:**

- Identify methodological heterogeneity, variations in stimulation paradigms.
- To describe developmental trends by age and neural patterns, if data permits.
- To identify clinical relevance and prognostic value when analyzing sMMN in children (<18 years).
- Provide recommendations for methodological approaches in future research protocols.

## 3. Materials and Methods

A systematic literature search was conducted between 17 and 18 August 2025, in the following database: PubMed (11), Scopus (6), Web of Science (6), DOAJ (1), Europe PMC (6), Embase (0), ClinicalKey (0), Cochrane Library (0) and ClinicalTrials.gov (0), resulting in a total of 30 articles. Search terms included: “mismatch negativity somatosensory”, “somatosensory mismatch negativity”, “tactile mismatch negativity”, “somatosensory ERP”, “tactile ERP” combined with “child”, “children”, “adolescent”, “adolescents”, “pediatric”, “infant” or “newborn”. Forward and backward citation tracking was performed, but no relevant additional studies were identified. Search protocol is available in the Supplementary Materials. The risk of bias was evaluated with modified MMAT. The exclusion of gray literature was intended to enhance methodological consistency and quality, although it may have contributed to potential publication bias.

The review was initially designed following a systematic review approach; however, owing to the limited number and heterogeneity of included studies, quantitative synthesis was not feasible. Therefore, a narrative synthesis was undertaken, grounded in a structured and descriptive comparison of study characteristics and findings, while acknowledging the limited methodological heterogeneity, the paucity of investigations across diverse pathologies, and the absence of age-stratified studies that would permit a dynamic developmental perspective.

Studies were grouped thematically based on stimulus modality (pneumatic, electrical, vibrotactile), population characteristics (infants versus older children; healthy versus clinical populations), and experimental parameters such as stimulation site and paradigm structure. Within each thematic group, findings were compared in terms of sMMN characteristics, including amplitude, latency, and spatial distribution. Directionality assessment was conducted by evaluating reported sMMN responses, with more negative amplitudes interpreted as reflecting stronger neural activity. Differences between standard and deviant stimuli, as well as between stimulation locations, were used to assess the direction and consistency of effects across studies.

While the review aimed to identify clinical relevance and prognostic value of sMMN in children, the limited number of available studies precluded drawing firm conclusions. These aspects are therefore highlighted as key directions for future research.

The study selection process is presented in accordance with the PRISMA 2020 guidelines. The review was registered in PROSPERO (Registration number: CRD420251246737) to ensure transparency and compliance with reporting standards.

## 4. Inclusion and Exclusion Criteria

Studies were included if they met the following criteria:

- participants younger than 18 years (newborns, infants, children, and adolescents);
- use of the oddball paradigm to evaluate sMMN responses;
- original research articles, published in English, with full data available;
- clinical, single-case reports or experimental studies including both typically developing children and children with neurological conditions.

The characteristics of all included studies are presented in Table 1.

Studies were excluded if they had exclusivity for adult participants, were narrative reviews or editorials and reported incomplete methodology or provided insufficient data for critical appraisal.

## 5. Selection Process

**Figure 2:**
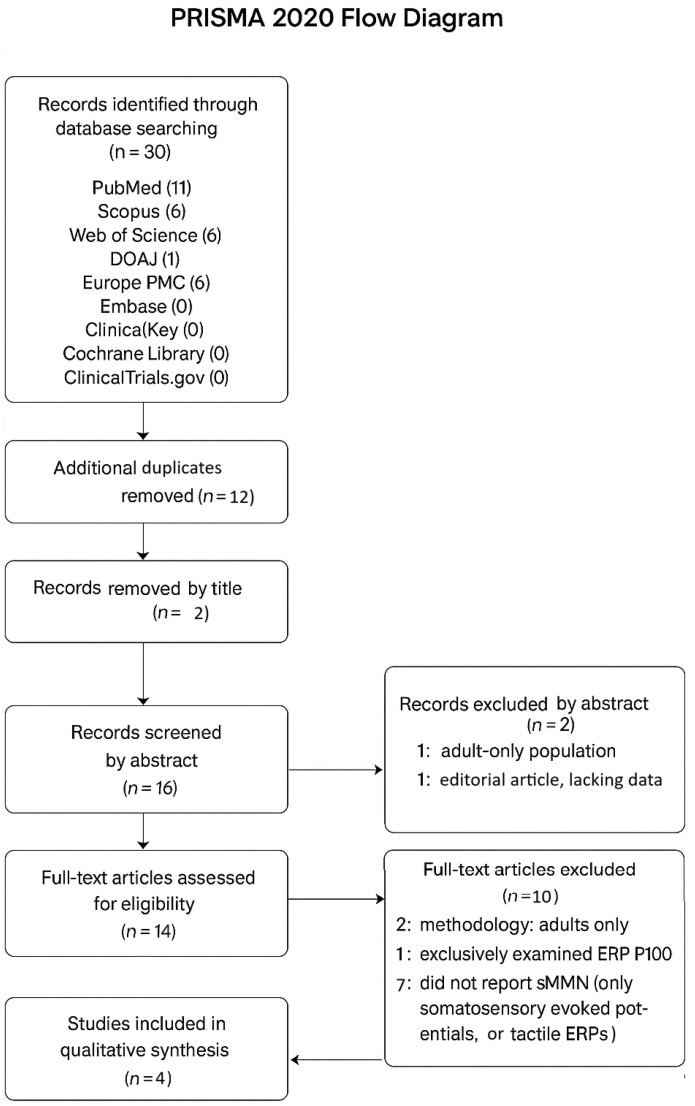
The PRISMA 2020 flow diagram illustrates the study selection process for the systematic review. A total of 30 records were initially identified across nine databases: (PubMed (11), Scopus (6), Web of Science (6), DOAJ (1), Europe PMC (6), Embase (0), ClinicalKey (0), Cochrane Library (0) and ClinicalTrials.gov (0)). After duplicate removal, 18 records remained for title screening, of which 2 were excluded. The remaining 16 records underwent abstract screening, leading to the exclusion of 2 studies (one involving only adult participants and one editorial lacking sufficient data). A total of 14 full-text articles were then assessed for eligibility. Of these, 10 were excluded for the following reasons: adult-only samples in methodology (*n* = 2), exclusive focus on the P100 component (*n* = 1), absence of sMMN data (*n* = 6), and reporting only tactile ERPs unrelated to sMMN (*n* = 1). Ultimately, 4 studies met all inclusion criteria and were included in the qualitative synthesis. Forward and backward citation tracking was performed, but no relevant additional studies were identified.

The study selection process was conducted in two stages. Initially, a total of 30 records were identified through database searches. After removing the duplicates, 18 records remained for title screening. During this stage, two articles were excluded based on the title, remaining 16 for abstract. Abstract screening resulted in another article being eliminated due to it studying only adults, and another one by being an editorial article with lack of data. Resulting in 14 articles that passed at the reading screening. During the full text screening, 10 articles were excluded. Two articles for mentioning studying only adults in methodology, and the other eight for studying exclusively the event-related potential P100 or for only mentioning somatosensory evoked potentials (SSEPs) not sMMN. Ultimately, only four studies were included in the systematic review as seen in Figure 2 of the **PRISMA Flow diagram**.

Screening and selection were performed independently by two reviewers. Discrepancies were resolved by discussion, with a third reviewer adjudicating any unresolved disagreements.

## 6. Data Extraction, Outcomes and Effect Measures

The study selection process is illustrated using a PRISMA flow diagram, showing the number of records identified, screened, excluded, and included at each stage. Extracted data included: age groups, pathology, stimulus type location and intensity, trial counts, application period, type of task, EEG channel montage, sMMN amplitude, peak, range amplitude, mean amplitude, latency window, distribution and deviant probability.

A narrative synthesis of sMMN characteristics and clinical relevance was performed. Comparisons of age groups, methodologies and developmental trends by age, was planned but not possible. Methodological gaps and heterogeneity were identified. Meta-analysis was not planned due to expected variability across studies. Subgroup analyses was not able to be conducted in order to examine potential differences in sMMN characteristics and clinical relevance across key variables considering the small number of articles.

**Outcomes:**

**Primary outcomes:** Stimulus type and location, age groups, trial counts, stimulus intensity, and application period, type of task, EEG channel montage, sMMN amplitude, peak, range amplitude, mean amplitude, latency window, distribution and deviant probability.
**Secondary outcomes:** Methodological heterogeneity, clinical relevance (e.g., prognostic value in autistic spectrum disorder, epilepsy), and developmental trends by age.

For the outcomes, the following **types of effect measures** were extracted:

**Continuous measures:** sMMN amplitude μV, peak amplitude, range amplitude, mean amplitude, latency window, stimulus intensity, trial counts, deviant probability.
**Comparative/categorical measures:** Stimulus type, stimulus location, age groups, task type, EEG montage, distribution, application period.
**Association-type measures:** Methodological heterogeneity, clinical relevance, prognostic value, developmental trends.

## 7. Quality Assessment

The risk of bias of the included studies was assessed using MMAT. All studies exhibited concerns regarding participant selection, control of confounders, and completeness of outcome reporting. These assessments are presented alongside the study results to provide context for the reliability of the findings.

The methodological quality was evaluated independently by two reviewers. Particular attention was given to the clarity of experimental paradigms and stimulation parameters, adequacy of sample size and participant characteristics, rigor of recording and preprocessing procedures for evoked potentials, and appropriateness of statistical analysis methods. Discrepancies between reviewers were resolved by consensus or arbitration by a third reviewer.

The outcomes of this quality assessment, summarized in Table 2, were incorporated into the narrative synthesis, with studies rated as high, moderate, or low quality explicitly noted to ensure a balanced and critical interpretation of the available literature, based on their MMAT scores (Q1–Q5: ✓/ × / ?). Common methodological concerns included small sample sizes, single-case reports, incomplete reporting of participant characteristics, insufficient detail on stimulation paradigms, and unclear outcome measurement procedures—particularly regarding sMMN extraction and preprocessing.

Because of these consistent limitations and methodological variability, the certainty of evidence regarding sMMN characteristics in pediatric and clinical populations remains low, and findings should be interpreted with caution. The risk of bias of the included studies was assessed using MMAT. These assessments are reported alongside the study results to provide context for the reliability of the findings.

## 8. Results

### 8.1. General Characteristics of Included Studies

A total of four studies investigating sMMN in pediatric populations (<18 years) were identified and included (Table 1). The studies originated from diverse geographical contexts, with sample sizes ranging from single-case reports, such as children with refractory epilepsy [16], to cohorts of approximately 25–30 participants [11,12,13]. The age of participants covered a wide developmental spectrum, from infants aged at 6 months [12,13] to adolescents [11].

All studies used passive oddball paradigms, using either electrical, pneumatic or vibrotactile stimulation [11,16], where children were either watching a silent video or engaged in a non-demanding task. This supports the view that sMMN primarily reflects pre-attentive cortical processing, independent of active attention or task engagement, making it suitable for pediatric populations who may have limited compliance.

Across the four included studies investigating somatosensory mismatch negativity (sMMN) in children, several consistent and divergent patterns emerge.

### 8.2. Methodological Heterogeneity and Its Impact on sMMN Measures

The studies employed a variety of somatosensory stimuli, including electrical tactile pulses [11], pneumatic tactile stimulation [12,13], and vibrotactile stimulation [16]. This variability indicates that no standardized stimulus protocol currently exists for pediatric sMMN research, limiting direct comparability across studies and highlighting the need for methodological consensus.

Considerable variability was also observed in experimental parameters, particularly regarding stimulation type, stimulus intensity, and number of trials. In infant populations, tactile stimulation was typically delivered using pneumatic devices with airflow pressures around 60 psi, whereas studies involving older children employed electrical stimulation of approximately 4 mA or vibrotactile stimuli. The number of trials ranged from approximately 1000 to 2000 stimuli, while the probability of deviant stimuli was generally set between 10% and 20% in oddball paradigms.

This methodological variability directly influences key sMMN characteristics, including amplitude and, to some extent, latency, thereby limiting cross-study comparability. Although these differences complicate direct quantitative comparisons between studies, they also demonstrate that somatosensory mismatch negativity (sMMN) can be reliably elicited across a variety of stimulation modalities and experimental conditions.

Amplitude modulation appears to be particularly sensitive to stimulus characteristics and the nature of the deviant event. In infant studies, larger amplitudes have been reported when stimuli represent categorical changes between different body regions, compared to deviations within the same anatomical category. This finding suggests that the somatosensory system may encode categorical body representations early in development. Moreover, sMMN amplitude is sensitive not only to the presence of a deviant stimulus but also to the magnitude of deviation, supporting its use as a quantitative marker of cortical sensory discrimination.

Furthermore, studies employing intracranial recordings have reported substantially larger amplitudes compared to scalp EEG recordings, likely reflecting the higher spatial resolution and improved signal-to-noise ratio associated with intracranial electrophysiological measurements.

### 8.3. Latency Differences Reflect Developmental Factors

Differences in latency across studies likely reflect developmental factors related to the maturation of neural processing. In infants [12], sMMN responses have been reported in earlier latency ranges, typically around 90–120 ms following stimulus onset [11,16]. In contrast, studies involving older children describe later components, with peaks occurring approximately between 160 and 220 ms.

These latency differences may reflect ongoing maturation of cortical networks, increased efficiency of thalamo-cortical connectivity, and progressive refinement of sensory predictive mechanisms. Developmental changes in synaptic organization and myelination may also contribute to the temporal dynamics of somatosensory processing observed across age groups, suggesting that sMMN latency may serve as a potential biomarker of neurodevelopmental progression in the somatosensory domain.

### 8.4. Detectability in Clinical and Typical Populations

The studies reviewed demonstrate that sMMN can be detected in both typically developing individuals and clinical populations. In healthy children, sMMN responses have been consistently observed during passive oddball paradigms involving tactile stimulation. Importantly, similar responses have also been recorded in clinical populations, such as children with medically refractory epilepsy undergoing intracranial monitoring.

The presence of sMMN in these clinical contexts suggests that the neural mechanisms responsible for deviance detection may remain functional even in the presence of neurological pathology. This supports the potential utility of sMMN as a tool for investigating sensory processing in both research and clinical settings.

### 8.5. Consistent Neural Patterns and Developmental Trends

Despite methodological heterogeneity, relatively consistent spatial and temporal patterns of sMMN emerge across studies. Most investigations report activation in frontal and fronto-central scalp regions, as well as centro-parietal areas contralateral to the stimulated body site, suggesting the involvement of a distributed cortical network that includes the primary somatosensory cortex and frontal regions implicated in deviance detection and predictive processing.

The convergence of these findings across diverse stimulation modalities and participant populations indicates that the neural mechanisms underlying somatosensory deviance detection are relatively stable across developmental stages. These observations align with adult literature and support the reliability of sMMN as a non-invasive measure of sensory prediction error in children.

## 9. Discussion

### 9.1. Methodological Heterogeneity and Developmental Trends in Pediatric sMMN

The analysis of the reviewed studies reveals notable methodological heterogeneity in pediatric sMMN research. Across studies, the types of stimuli used varied considerably, including electrical, pneumatic, and vibrotactile modalities. Deviants differed in intensity, location, and frequency, and interstimulus intervals ranged from 600 to 1000 ms. Furthermore, the number of trials and the proportion of deviant stimuli were inconsistent, with some studies presenting as few as 1000 stimuli and others up to 2000, while deviant probabilities varied between 10 and 20 percent. EEG recording setups were also heterogeneous, employing between 15 and 32 channels with differing reference schemes. These variations were shown to influence both sMMN amplitude and latency, which complicates cross-study comparisons and underscores the need for more standardized paradigms to improve reproducibility and allow meaningful meta-analytic integration.

**Figure 3:**
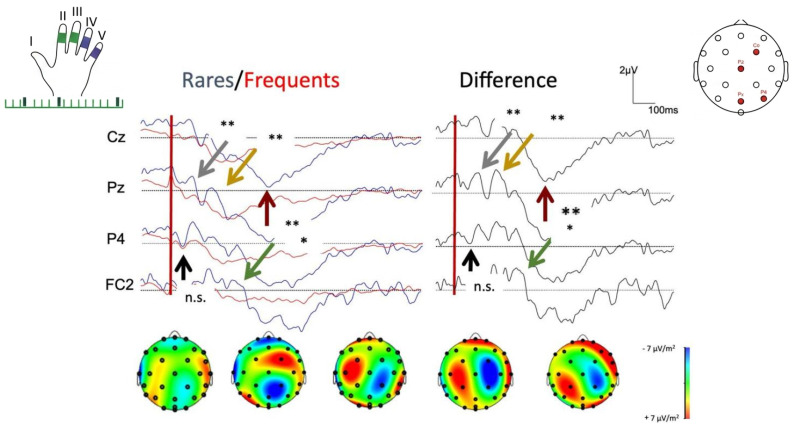
**Somatosensory Mismatch Negativity (sMMN) elicited by rare tactile stimuli.** Rare tactile stimuli elicit a clear somatosensory mismatch negativity (sMMN) compared with frequent stimuli, with maximal responses at 60–250 ms over central–parietal regions. **Tactile stimulation (upper left corner):** The hand illustration shows digits II and III as frequent (standard) stimuli and digits IV and V as rare (deviant) stimuli. The line below represents the stimulation sequence: frequent stimuli (green) and rare stimuli (purple). **Electrode map (upper right corner):** Head diagram with the electrode positions used for analysis (Cz, Pz, P4, FC2). **EEG responses**—Electrodes Cz, Pz, P4, FC2 (center graphs): The column “Rares/Frequents” shows somatosensory evoked potentials for rare (blue) and frequent (red) stimuli. The column “Difference” shows the subtraction (rare–frequent), highlighting the sMMN component. Red vertical line (0 ms) indicates the stimulus onset. Black dotted lines represent baseline. **Colored arrows:** Gray and yellow (Cz, 60–140 ms) → early N1/P2 somatosensory responses. Red (Pz, 140–250 ms) → characteristic negativity of sMMN (differential response to rare vs frequent). Black (P4, very early, 45 ms) → initial response, not significant (n.s.). Green (FC2, 250 ms) → additional significant activation. Statistical markers: * and ** = statistically significant (* = *p* < 0.05, ** = *p* < 0.01). “n.s.” = not significant. **Scalp maps (bottom):** Show the spatial distribution of cortical activity at different post-stimulus latencies (45, 60, 140, 250, 400 ms). Red/yellow = positive activations, blue = negative activations. Strongest differential response (sMMN) occurs at 60–250 ms, central–parietal distribution. **Original data** obtained by the senior author (AB) from a patient using Micromed brain quick, Rome, Italy.

The importance of these parameters is illustrated in Figure 3, which provides a representative example of sMMN elicited using a tactile oddball paradigm, highlighting differences between rare and frequent stimuli in terms of amplitude, latency, and spatial distribution.

This methodological variability complicates the interpretation of developmental trends, as differences in stimulation parameters and recording setups may partially account for the variability observed in sMMN latency and amplitude across age groups. Despite these limitations, consistent developmental patterns were observed, with sMMN latency generally increasing with age. Infants typically exhibited shorter latencies around 60–120 ms, while older children showed longer latencies of approximately 135–220 ms. Amplitude tended to increase with maturation, reflecting greater cortical specialization, and the cortical distribution of sMMN became more focal over frontocentral regions as children grew older. These patterns suggest that sMMN may serve as a non-invasive index of somatosensory system maturation, providing insights into both cortical development and the efficiency of sensory discrimination.

### 9.2. Clinical Relevance and Prognostic Value

Although methodological variability and developmental factors complicate interpretation, emerging evidence suggests potential clinical relevance. Despite the predominance of studies on typically developing children, some included clinical populations, such as children with refractory epilepsy. These studies suggest that sMMN can be detected even in the context of neurological disorders and that deviations in amplitude or cortical distribution may reflect atypical somatosensory processing. This indicates that sMMN has the potential to serve as a prognostic biomarker for developmental or sensory processing impairments. Although current evidence remains preliminary due to small sample sizes, the findings highlight the translational relevance of pediatric sMMN in identifying early sensory deficits and potentially guiding interventions.

### 9.3. Comparison with Studies on Adults

Somatosensory mismatch negativity (sMMN) has been relatively well studied as an event-related potential in adults, reflecting automatic detection of tactile deviance within predictive-processing frameworks. Numerous studies have demonstrated consistent sMMN responses in healthy adults, typically appearing as fronto-central or centro-parietal negativities between 150 and 300 ms [23]. For instance, Akatsuka et al. (2005) [23] described two characteristic latency windows (180–220 ms and 250–290 ms), while recent vibrotactile paradigms reported reliable sMMN for duration deviants differing from the standard by ≥30 ms. These findings confirm a stable neural mechanism involving somatosensory and frontal generators.

Comparative pediatric data show similar patterns. In a foundational study of typically developing children aged 6–11 years, sMMN emerged at approximately 160 ms (left-central negativity) followed by a frontal negative response around 220 ms, using an electrical oddball paradigm with non-nociceptive stimuli (standard: thumb, 80%; deviant: fifth finger, 20%) and passive attention (children distracted by a video game) [11]. These parameters—high-probability standards, low-probability deviants, passive attention—are comparable to those used in adult protocols, supporting meaningful developmental comparisons. The close similarity in latency and topography suggests that the automatic somatosensory change-detection system is already functional by middle childhood.

Taken together, adult studies offer essential benchmarks for interpreting pediatric sMMN and support its potential role as a developmental biomarker for somatosensory processing.

### 9.4. Recommendations for Future Research Protocols

Based on these observations, future research should contain: age groups, pathology, stymulus type location and intensity, EEG number of electrodes, trial counts and blocks, apllication period, distribution, amplitude of sMMN, range amplitude and peak, latency, probabilities (standard and deviant), extraction method, and task type. Age-stratified analyses should be included to contextualize developmental changes in latency and amplitude, it is essential that studies expand to include a broader range of clinical data, and longitudinal designs are encouraged to track sensory system maturation and the effects of interventions over time.

Nevertheless, sMMN is of particular interest because it reflects the brain’s detection of unexpected tactile events occurring on the body as seen in Figure 3.

In summary, the secondary outcomes highlight considerable methodological variability in pediatric sMMN research, while also revealing consistent developmental trends and potential clinical utility. Addressing these methodological challenges and incorporating larger, age-stratified, and clinically diverse samples will be essential to establish sMMN as a reliable biomarker of somatosensory processing and neurodevelopment in children.

## 10. Limitations of the Existing Literature and Methodology

Taken together, current evidence suggests that sMMN has potential as a pediatric clinical marker. However, the existing studies present several limitations. Sample sizes are generally small, limiting statistical power and generalizability [5,11,16]. Methodological heterogeneity—including variations in stimulus paradigms, recording techniques, and analysis approaches—complicates cross-study comparisons and the establishment of normative data [11,12,13]. Additionally, most research focuses on narrow clinical populations, often lacking age-matched healthy controls, which restricts the interpretation of developmental trajectories [12,13]. Moreover, crucial methodological details such as session duration, ISI, and deviant probabilities, limit the generalizability of findings. These factors highlight the necessity of harmonized protocols for pediatric sMMN studies to enable meta-analytic comparisons and longitudinal investigations.

Furthermore, reporting standards are inconsistent, and potential confounding factors such as attention, medication, or comorbidities are not always controlled [11,12,13]. Additionally, limited data on children with neurodevelopmental conditions such as ASD restricts the generalizability of findings beyond typically developing or severe neurological populations.

This systematic review is subject to certain methodological limitations. The search strategy was comprehensive and highly inclusive, maximizing the likelihood of identifying all relevant studies, although the possibility of missing some relevant studies cannot be entirely excluded. Gray literature was not searched. The exclusion of gray literature was intended to enhance methodological consistency and quality, although it may have contributed to potential publication bias. Additionally, the small number of available studies and variability in study designs precluded quantitative synthesis and limited the ability to perform subgroup analyses. The high and heterogeneous risk of bias identified with MMAT further emphasizes the need for cautious interpretation of the findings. These factors should be considered when interpreting the findings. Finally, incomplete reporting of sMMN latency and amplitude values in infants and younger children limits the precision of developmental comparisons and the establishment of normative data.

## 11. Future Research Directions

To strengthen the role of sMMN as a pediatric neurophysiological marker, future research should prioritize the standardization of stimulation paradigms, recording parameters, analytic procedures, and detailed methodological reporting—including stimulus intensity, deviant probability, number of blocks and trials, and temporal analysis windows. Such standardization would facilitate cross-study comparisons, enable subgroup analyses by age, pathology, and recording method, and support the establishment of normative benchmarks.

Expanding studies to larger and age-stratified cohorts would enhance statistical power, generalizability, and the characterization of developmental trajectories, from infancy to adolescence, in terms of latency, amplitude, cortical sources, and individual variability. Longitudinal investigations are essential to track sMMN maturation, relate it to cortical plasticity, and clarify its role in neurocognitive development.

Inclusion of diverse clinical populations, including children with neurodevelopmental and neuropsychiatric disorders such as ASD, as well as children recovering from coma or hypoxic–ischemic encephalopathy, is crucial to validate the clinical utility and prognostic value of sMMN as a diagnostic and prognostic biomarker, and to enable the establishment of standardized protocols that would improve comparability, reproducibility, and clinical translation across studies.

Finally, several studies lacked complete reporting of secondary outcomes, such as correlations with behavioral measures, sensory profiles, or cognitive indices, and in some cases, crucial details like ISI or stimulus intensity were not consistently reported, leading to gaps in the interpretation of results.

Integrating sMMN data with multimodal approaches, such as neuroimaging and comprehensive behavioral measures, will improve mechanistic understanding, refine prognostic predictions, and support translation into clinical practice.

## 12. Conclusions

This review suggests that evidence on somatosensory mismatch negativity (sMMN) in children remains limited, heterogeneous, and largely non-standardized. Nevertheless, existing studies consistently demonstrate that sMMN can be reliably elicited in pediatric populations, including both typically developing children and those with neurological or neurodevelopmental disorders, indicating that automatic tactile change detection mechanisms are present early in life.

However, substantial methodological variability exists across studies, particularly in stimulation paradigms, interstimulus intervals, EEG acquisition systems, and regions of interest, which may account for differences in reported amplitudes and latencies. Although clinical applications remain exploratory, altered sMMN responses appear to reflect atypical somatosensory processing in conditions such as autism spectrum disorder and epilepsy, suggesting potential value as a biomarker of sensory processing abnormalities.

Preliminary findings also point toward developmental maturation of sMMN responses with age, though current evidence is insufficient to define normative trajectories. Overall, future research should focus on standardized experimental protocols, larger age-stratified cohorts, and longitudinal designs to better characterize developmental patterns and clarify the clinical utility of sMMN in pediatric populations.

## Figures and Tables

**Table 1:** The characteristics of the studies that were investigating sMMN in pediatric populations (<18 years) and were included.

Characteristic	Shen et al. (2020) [13]	Shen et al. (2018) [12]	Restuccia et al. (2009) [11]	Spackman et al. (2010) [16]
Age groups	6–7 m	6–7 m	6–11 y	6–17 y
Pathology	Healthy	Healthy	Healthy	Refractory epilepsy
Stimulus Type	Pneumatic	Pneumatic	Electric	Vibrotactile
Stimulus Location	Hand vs. forearm	Right cheek vs. right hand/neck	Right thumb (S) vs. right 5th finger (D)	Distal phalanx digits 2 and 3
Stimulus Intensity	60 psi airflow pressure	60 psi airflow pressure	3.5 mA to 4.5 mA	+/− 1.9 V (T-bar displacement)
EEG Montage	32E/10–20 format	32E/10–20 format	15E	intracranial electrodes
Trial Counts	1000 (80%S/20D)	1000 (80%S + 10D hand + 10D neck)	1000 (80%S/20D)	2000 (90%S/10D)
Probability	S80 + D10	S80 + Dh10 + Dn10	S80 + D20	S90 + D10
Task Type	Passive	Passive	Passive	Passive
Application Period	SD100 ms + ISI 600 ms	SD100 ms + ISI 600 ms	ISI 1000 ms	SD20 ms/250 ms + ISI 1000 ms
Distribution sMMN	F + Fc + C	F + Fc + Tc	Cp + F	Pc + Pf
sMMN Amplitude	Forearm: −3.4 to Hand: −5.1 μV	Neck stimulation: −6.09 μV Hand stimulation: −5.08 μV	NA	−21.75 +/− 6.23 μV
Range Amplitude	−5.1 to −3.4 μV	H: −3.18 to N: −6.09 μV	NA	−25.56 to −85.39 μV
Peak Amplitude	NA	Hand: −3.18 μV to Neck: −4.1 μV	120–180 ms and 180–250 ms	21.75 +/− 6.23 μV
Latency	60–180 ms	60–180 ms	160 and 220 ms	135 +/− 13 ms

**Table legend**: m—months; y—years; NA—not available; E—electrodes; ISI—interstimulus interval; F—frontal; Fc—fronto-central; C—central; D—deviant; Dh—deviant hand; Dn—deviant neck; S—standard; SD—stimulus duration; Cp—centro-parietal; Pc—postcentral; Pf—prefrontal; Tc—temporo-central.

**Table 2:** The results of the quality assessment: a simplified summary of MMAT assessment categories, showing the risk of bias for each domain in each study.

Study	Study Design	Q1-P	Q2-M	Q3-C	Q4-O	Q5-A	Score
Shen et al. (2020) [13]	Cantitative descriptive	✓	✓	?	✓	✓	4/5
Shen et al. (2018) [12]	Cantitative descriptive	✓	?	✓	✓	✓	4/5
Restuccia et al. (2009) [11]	Experimental (oddball and standard-omitted)	×	✓	?	✓	✓	3/5
Spackman et al. (2010) [16]	Quantitative non-experimental, cross-sectional, comparative groups	✓	✓	?	✓	✓	4/5

**Table legend**: Q1-P (Population): Is the sampling strategy relevant to address the research question?; Q2-M (Measurement): Are the measurements appropriate and well-defined?; Q3-C (Confounders): Are confounders accounted for?; Q4-O (Outcome): Is the risk of nonresponse bias low?; Q5-A (Analysis): Is the statistical analysis appropriate to answer the research question?; ? = Cannot tell. ✓ = The article meets the criterion. × = The article does not meet the criterion.

## Data Availability

No new data were created or analyzed in this study. Data sharing is not applicable to this article.

## Supplementary Materials

The following supporting information can be downloaded at: https://www.mdpi.com/article/10.3390/diagnostics16101471/s1. File S1: Search Strategy for Review: sMMN in Children. File S2: PROSPERO Protocol.

## Abbreviations

sMMN	somatosensory mismatch negativity
aMMN	auditory mismatch negativity
vMMN	visual mismatch negativity
MMN	mismatch negativitiy
ERP	event-related potential
SEP	somatosensory evoked potential
AEP	auditory evoked potential
EEG	electroencephalogram
MEG	magnetoencefalogram
MMAT	mixed methods appraisal tool
DOAJ	Directory of Open Access Journals
ms	milliseconds
Fz/F1/F…	Frontal, it refers to the localization of electrodes on the head
Cz/C1/C…	Central, it refers to the localization of electrodes on the head
Oz/O1/O…	Occipital, it refers to the localization of electrodes on the head
Pz/P1/P…	Parietal, it refers to the localization of electrodes on the head
FC1/FC2/FC…	Fronto-central, it refers to the localization of electrodes on the head
n.s.	not significant
N1	component of event-related potentials, at 100 ms, early sensory detection.
N20	component of event-related potentials, at 20 ms, very early somatosensory response.
N200	component of event-related potentials, at 200 ms, deviance processing; includes the MMN.
P100	component of event-related potentials, at 100 ms, early positive component.
P300	component of event-related potentials, at 300 ms, attentional orienting and cognitive processing.
