# Supplementary material for: Somatosensory Mismatch Negativity in Children: A Narrative Review of Current Evidence and Methodological Considerations
Source: Diagnostics (Basel). 2026 May 12;16(10):1471. doi: 10.3390/diagnostics16101471 (PMC13205875; doi:10.3390/diagnostics16101471)
Supplement: Supplementary file 1 [file diagnostics-16-01471-s001.zip › PROSPERO protocol.pdf]

PROSPERO Protocol

Title: Somatosensory Mismatch Negativity in Children: A Narrative Review of Current Evidence and Methodological Considerations

Review type: Narrative review

Author for correspondence:
- **Name:** Nussbaum Laura Alexandra
- **Affiliation:** Department of Neurosciences within the Neuroscience Research Center at Victor Babes University of Medicine and Pharmacy, Timisoara, Romania
- **Email:** nussbaum.laura.alexandra@gmail.com

Other authors:
- **Name:** Brinzeu Andrei
- **Affiliation:**
    - Department of Neurosciences within the Neuroscience Research Center at Victor Babes University of Medicine and Pharmacy, Timisoara, Romania
    - Neuropain Unit, Lyon Neuroscience Research Center (CRNL), Claude Bernard University Lyon 1, CETD Hospices Civils de Lyon, France

- **Name:** Ardelean Adelina Amalia
- **Affiliation:** Department of Neurosciences within the Neuroscience Research Center at Victor Babes University of Medicine and Pharmacy, Timisoara, Romania

Dates:
- **Search period:** 17–18 August 2025
- **Completion date:** 3 January 2025

1. Review question / Objectives

Primary objective:

To systematically evaluate the characteristics, developmental trajectory, and clinical relevance of somatosensory mismatch negativity (sMMN) in children (<18 years), including latency, amplitude, topography, and prognostic applications.

Secondary objectives:

- Identify methodological variations in stimulation paradigms and recording techniques.
- Assess sMMN as a potential biomarker for pediatric neurological and neurodevelopmental conditions.
- Provide guidance for future research protocols.

2. Participants / Population

- Children aged 0–18 years (newborns, infants, children, adolescents)
- Healthy participants and pediatric patients with neurological or neurodevelopmental conditions

3. Intervention / Exposure

Somatosensory stimulation (tactile, electrical, vibrotactile) using oddball paradigms EEG (32–64 channels) or intracranial recordings for sMMN

4. Comparator / Control

Healthy children or baseline standard stimuli (frequent vs deviant)

5. Outcomes

Primary outcomes:

- sMMN latency (ms)
- application period
- Topographical distribution
- Developmental trends by age

Secondary outcomes:

- Methodological heterogeneity
- Clinical relevance (e.g., prognostic value in autistic spectrum disorder, hypoxic– ischemic encephalopathy, epilepsy)
- Relationship with SEPs and AEPs

6. Types of studies

- Original research articles (experimental or clinical)
- Published in English, with full data available
- Excludes narrative reviews, editorials, and insufficient methodology

7. Information sources / Search strategy

- Databases: PubMed, Scopus, Web of Science, DOAJ, Europe PMC, Embase, ClinicalKey, Cochrane Library and ClinicalTrials.gov
- Keywords: "mismatch negativity somatosensory", "somatosensory mismatch negativity", "tactile mismatch negativity", "somatosensory ERP", "tactile ERP" combined with

"child", "children", "adolescent", "adolescents", "pediatric", "infant" or "new-born".
- Language: English Screening of titles/abstracts, followed by full-text review

8. Inclusion and exclusion criteria

Inclusion:

- Participants <18 years
- Oddball paradigm used for sMMN evaluation
- Original research, English language, full data

Exclusion:

- Adult-only studies
- Narrative reviews, editorials
- Incomplete or insufficient data

9. Data extraction

- By two independent reviewers
- Data items: sample size, age, pathology, stimulation parameters, EEG method, application period, sMMN latency, topography, clinical relevance
- Discrepancies resolved by consensus

10. Risk of bias / Quality assessment

- Articles were screened for relevance to sMMN metrics (stimulation parameters, latency, methodology)
- Editorials and studies without usable sMMN data were excluded
- Was made with MMAT - mixed methods appraisal tool

11. Data synthesis / Analysis

- Narrative synthesis of sMMN characteristics and clinical relevance
- Comparison across age groups, pathologies, and methodologies
- Identification of methodological gaps and heterogeneity
- Meta-analysis **not planned** due to heterogeneity

12. Subgroup analyses (if data allow)

- By age group (infants, preschool, school-aged, adolescents)
- By neurological condition (e.g. autism spectrum disorder, hypoxic–ischemic encephalopathy,)
- By stimulus modality (electrical, vibrotactile)
- By recording method (EEG vs intracranial)

13. Dissemination plan

- Publication in peer-reviewed pediatric neurology / neuroscience journals
- Presentation at international conferences

14. Registration

- Submission to PROSPERO for transparency
