# Supplementary material for: Somatosensory Mismatch Negativity in Children: A Narrative Review of Current Evidence and Methodological Considerations
Source: Diagnostics (Basel). 2026 May 12;16(10):1471. doi: 10.3390/diagnostics16101471 (PMC13205875; doi:10.3390/diagnostics16101471)
Supplement: Supplementary file 1 [file diagnostics-16-01471-s001.zip › Search Strategy for Review sMMN in Children.pdf]

Search Strategy for Review: sMMN in Children

1. **PudMed**

("somatosensory mismatch negativity" OR "sMMN" OR "tactile mismatch negativity" OR "somatosensory ERP" OR "tactile ERP")

AND

(child OR children OR adolescent OR adolescents OR pediatric OR infant OR newborn)

2. **Web of Science**

TS=("somatosensory mismatch negativity" OR "sMMN" OR "tactile mismatch negativity" OR "somatosensory ERP" OR "tactile ERP")

AND

TS=("child*" OR "children" OR "adolescent*" OR "adolescents" OR "pediatric*" OR "infant*" OR "newborn*")

3. **Scopus**

TITLE-ABS-KEY("somatosensory mismatch negativity" OR "sMMN" OR "tactile mismatch negativity" OR "somatosensory ERP" OR "tactile ERP")

AND

TITLE-ABS-KEY(child* OR children OR adolescent* OR adolescents OR pediatric* OR infant* OR newborn*)

4. **DOAJ**

("somatosensory mismatch negativity" OR "sMMN" OR "tactile mismatch negativity" OR "somatosensory ERP" OR "tactile ERP")

AND

(child OR children OR adolescent OR adolescents OR pediatric OR infant OR newborn)

5. **Europe PMC**

("somatosensory mismatch negativity" OR "sMMN" OR "tactile mismatch negativity" OR "somatosensory ERP" OR "tactile ERP")

AND

(child OR children OR adolescent OR adolescents OR pediatric OR infant OR newborn)

6. **Embase**

('somatosensory mismatch negativity' OR 'sMMN' OR 'tactile mismatch negativity' OR 'somatosensory ERP' OR 'tactile ERP')

AND

('child'/exp OR 'children' OR 'adolescent'/exp OR 'adolescents' OR 'pediatric' OR 'infant'/exp OR 'newborn')

7. **ClinicalKey**

("somatosensory mismatch negativity" OR "sMMN" OR "tactile mismatch negativity" OR "somatosensory ERP" OR "tactile ERP")

AND

(child OR children OR adolescent OR adolescents OR pediatric OR infant OR newborn)

8. **Cochrane Library**

("somatosensory mismatch negativity" OR "sMMN" OR "tactile mismatch negativity" OR "somatosensory ERP" OR "tactile ERP")

AND

(child OR children OR adolescent OR adolescents OR pediatric OR infant OR newborn)

9. **ClinicalTrial.gov**

("somatosensory mismatch negativity" OR "sMMN" OR "tactile mismatch negativity" OR "somatosensory ERP" OR "tactile ERP")

AND

(child OR children OR adolescent OR adolescents OR pediatric OR infant OR newborn)

Forward and backward citation tracking was performed, but no relevant additional studies were identified.
